# The Role of Endocannabinoids in Physiological Processes and Disease Pathology: A Comprehensive Review

**DOI:** 10.3390/jcm14082851

**Published:** 2025-04-21

**Authors:** Paulina Simankowicz, Joanna Stępniewska

**Affiliations:** Department of Nephrology, Transplantology and Internal Medicine, Pomeranian Medical University, Powstancow Wielkopolskich 72, 70-111 Szczecin, Poland

**Keywords:** anandamide, 2-arachidonoylglycerol, cannabinoid receptors

## Abstract

The endocannabinoid system is a complex communication system involved in maintaining homeostasis in various physiological processes, including metabolism, immune response, pain modulation, and neuroprotection. Endocannabinoids, mainly anandamide and 2-arachidonoylglycerol, are natural ligands of the cannabinoid receptors CB1 and CB2, which are widely distributed throughout the central nervous system and peripheral tissues. Their biosynthesis, degradation, and interaction with other signaling pathways play crucial roles in both health and disease. This article provides a comprehensive overview of the physiological and pathological roles of endocannabinoids, discusses their potential as therapeutic targets, and highlights recent advances in endocannabinoid-based treatments.

## 1. Introduction

**Figure 1:**
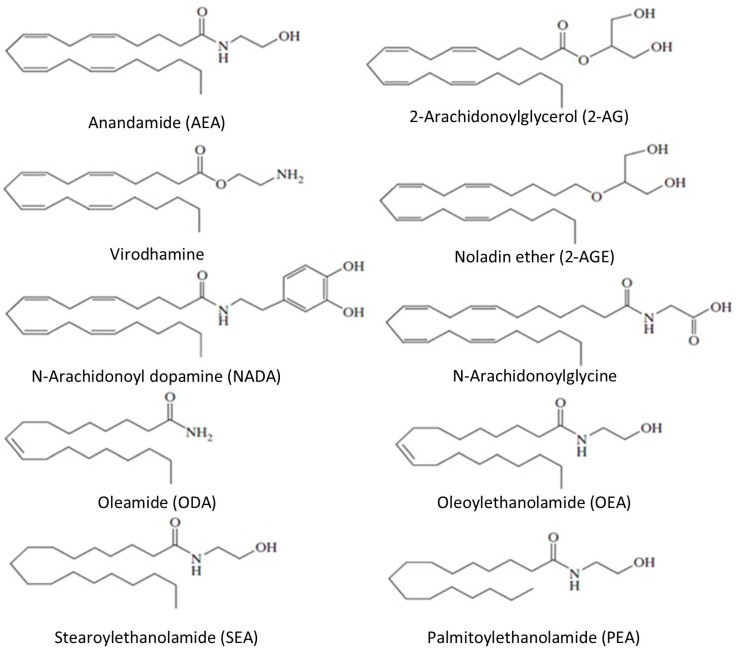
The chemical structure of endocannabinoids.

Endocannabinoids are derivatives of omega-6 polyunsaturated fatty acids. They are produced in the cell membranes of postsynaptic neurons from membrane phospholipids with the participation of phospholipase C. Endocannabinoids are part of the intercellular communication systems and show similarities to classical neurotransmitters. However, unlike them, they have a lipid structure, so they are not water soluble or stored in synaptic vesicles [1,2]. Their biosynthesis and release occur under the influence of depolarization and the influx of calcium ions. They act as retrograde neurotransmitters, inhibiting the release of neurotransmitters such as GABA, glutamic acid, serotonin, and noradrenaline [3,4]. The most thoroughly studied endocannabinoids are anandamide (AEA) and 2-arachidonoylglycerol (2-AG). The group of endocannabinoids also includes other compounds that can bind to cannabinoid receptors: noladin ether (2-AGE), arachidonic acid ester with ethanolamine (virodhamine), N-arachidonoyl dopamine (NADA), oleamide (ODA), N-arachidonoyl glycine (NAGLy), as well as the N-acylethanolamines (NAE), which are derivatives of various fatty acids: palmitic (PEA), stearic (SEA), and oleic (OEA) [5]. See Figure 1.

**Figure 2:**
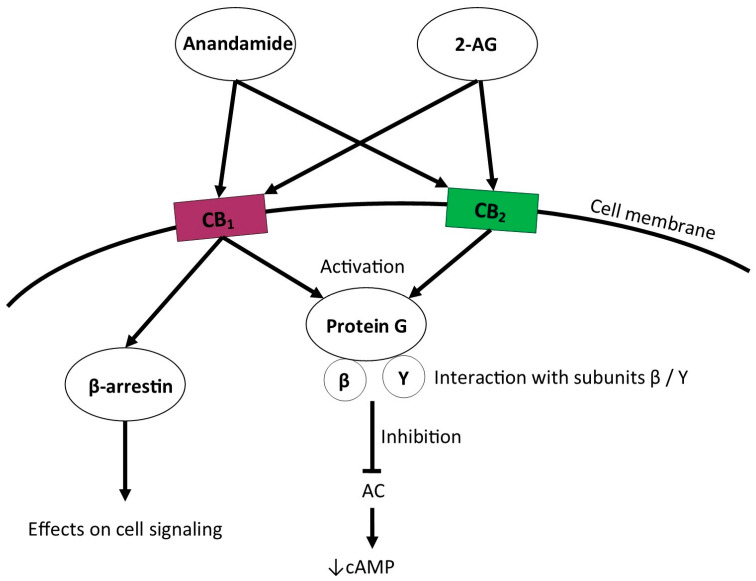
Signaling pathways induced by endocannabinoid receptors activation. 2-AG—2-arachidonylo-glicerol, CB1—cannabinoid receptor 1, CB2—cannabinoid receptor 2, AC—Adenylyl cyclase, cAMP—cyclic adenosine 3′,5′-monophosphate.

Anandamide, like 2-arachidonoylglycerol, transmits signals through CB1 and CB2 cannabinoid receptors. Endogenous ligands bind to cannabinoid receptors to activate the Gi/o protein, which then interacts with the β and γ subunits, initiating further signaling [5]. The primary effect is inhibition of adenylate cyclase (AC), which results in decreased cytosolic cyclic adenosine monophosphate (cAMP) levels [1]. Previous studies suggest that these receptors can also activate other proteins such as mitogen-activated kinases (MAPKs), including ERK, JNK, and p38MAPK [1,6]. Activation of the CB1 receptor also releases β-arrestin, which then prevents further G-protein binding of the receptor and leads to signal silencing [3,7]. Some cannabinoid ligands target β-arrestin signaling instead of cAMP signaling. β-Arrestin signaling leads to the suppression of the signal transmitted by the receptor via its desensitization or internalization, and it may produce specific effects on cells that can potentially be used therapeutically [6]. See Figure 2.

It is believed that endocannabinoids are not stored in cells but are mainly produced “on demand” during the synthesis of membrane phospholipid precursors [8]. However, there are reports that anandamide may be stored in the structures of cells synthesizing and storing simple fats [6,7,8].

The cannabinoid receptors CB1 and CB2 are involved in many physiological processes in the body [5,6]. CB1 receptors are located mainly in the brain and central nervous system but also in peripheral tissues such as muscles, liver, and adipose tissue. CB2 receptors are mainly associated with the immune system and have anti-inflammatory effects [6,7,9]. CB1 receptors are most abundant in the hypothalamus and pituitary gland. Their activation regulates all hypothalamic–pituitary axes [10].

CB1 receptors are located primarily on presynaptic terminals, whereas CB2 receptors can be found mainly in postsynaptic membranes [1]. Their stimulation inhibits adenylate cyclase activity, blocks calcium channels, increases the conductance of potassium channels, and activates mitogen-activated protein kinases (MAPKs) [3,5,11].

The end result is the inhibition of the release of many neurotransmitters such as acetylcholine, dopamine, serotonin, glutamate, and GABA [12]. There are also lesser-known receptors that can bind to cannabinoid compounds, such as transient receptor potential vanilloid 1 (TRPV1) and G protein-coupled receptor 55 (GPR55) [6,7,13].

Recently, much attention has been paid to the ability of the endocannabinoid system to control appetite, food intake, and energy balance, especially in light of the various mechanisms underlying these functions. Interactions with type 1 cannabinoid receptors are mainly associated with the regulation of appetite, motivation to eat, and pain perception. CB1 receptors have been described to be present throughout the enteric nervous system, where they act to inhibit both motility and secretion [14]. Interaction with type 2 cannabinoid receptors is primarily associated with the regulation of immune response processes [7].

It has been shown that disruption of homeostasis in the endocannabinoid system can cause the progression of many pathological conditions, including those affecting the cardiovascular system, central nervous system, kidneys (progression of nephropathy), adipose tissue (development of insulin resistance, obesity), liver, pancreas, bones, eyes, reproductive system, respiratory tract, and skin [8,9]. The cannabinoid system acts centrally through its effects on hypothalamic and mesolimbic appetite-regulating neurons and peripherally by influencing the function of adipocytes, hepatocytes, and the endocrine part of the pancreas [14]. See Table S1 in Supplementary Materials.

### 1.1. The Physiological Role of the Endocannabinoid System in Peripheral Tissues

In the study by Cota et al. using mouse models, the presence of CB1 receptors in adipocytes was demonstrated [15]. The function of CB1 receptors for 2-AG and AEA in the regulation of adipogenesis, lipogenesis and fatty acid utilization in adipose tissue, skeletal muscle, and the liver has been documented. Activation of CB1 receptors stimulates lipogenesis, reduces lipolysis, and accumulates triglycerides. After the use of CB1 receptor inverse agonists, the opposite effects of treatment were observed as well as a decrease in the levels of proinflammatory markers [16,17]. The production of endocannabinoids in adipocytes is under the negative control of insulin, and their increased level is associated with the development of insulin resistance [18]. The endocannabinoid system also participates in the regulation of adipocyte differentiation [18,19]. The study by Fanelia et al. conducted on a group of 121 patients who met strict inclusion criteria showed that higher plasma 2-AG levels in men and higher AEA levels in women are correlated with obesity and various metabolic parameters [20]. In summary, in the future, endocannabinoids can be considered as biomarkers of insulin resistance and the accumulation of intraabdominal white adipose tissue in the case of 2-AG and subcutaneous in the case of AEA. Further studies on obesity and assessment of whether plasma 2-AG levels can be used as a therapeutic target or as an indicator of lifestyle improvement and the effectiveness of pharmacological treatment are required.

### 1.2. The Physiological Role of the Endocannabinoids in the Central Nervous System

Endocannabinoids can be detected from the earliest stages of pregnancy, i.e., in the embryo, uterus, placenta, and developing fetal brain. In human fetal brains, CB1 receptors can be detected as early as 14 weeks of gestation, with the highest expression in the cerebral cortex, caudate nucleus, putamen, hippocampus, and cerebellar cortex. The location of CB1 receptors in the fetal central nervous system (CNS) mirrors their distribution in adults [21]. The CB1 cannabinoid receptor is the most abundant G protein-coupled receptor in the CNS. CB1 cannabinoid receptors affect cell signaling through the release of neurotransmitters. Studies to date have shown that the CB1 receptor plays a neuroprotective role, likely by influencing the regulation of neurotransmission via inhibition of GABA and glutamate release [22,23].

## 2. Materials and Methods

The aim of the review was to summarize the current knowledge on the role of endocannabinoids in physiological processes and disease pathology. A comprehensive literature review was conducted in the MEDLINE (PubMed) database in the period December 2024–February 2025. The list included original and review articles published in peer-reviewed journals. An effort was made to apply the publication date limitation within a maximum period of 10 years. The aim was to holistically collect the current knowledge on endocannabinoids, which is why publications exceeding the assumed time period were occasionally used. No additional search filters were added. When searching for articles, phrases such as “endocannabinoid system” or “endocannabinoids” in combination with “neurological disorders”, “psychiatric disorders”, “kidney diseases”, “glucose metabolism”, “gastrointestinal diseases”, “liver diseases”, “reproductive system”, and “asthma” were used.

## 3. The Role of the Endocannabinoid System in Disease Pathology

### 3.1. The Regulation of Glucose Homeostasis

It has been shown that the endocannabinoid system, through the activity of the CB1 receptor, influences the metabolic processes of adipose tissue and skeletal muscles and is involved in the processes of glucose utilization. Activation of CB1 receptors present in adipose tissue results in a decrease in the secretion of adiponectin, which has anti-inflammatory effects and sensitizes peripheral tissues to insulin [24,25,26]. Additionally, it is believed that endocannabinoids are natural ligands for peroxisome proliferator-activated receptors (PPARs), suggesting the involvement of cannabinoids in fatty acid metabolism [27]. It has been shown that pharmacological blockade of the CB1 receptor results in a temporary reduction in appetite, weight loss, and reduction in adipose tissue, and leads to numerous metabolic and hormonal changes expressed by decreased levels of leptin, insulin, glucose, and triacylglycerols in the blood, and increased adiponectin levels [25]. The involvement of endocannabinoids in autoimmune diseases, including diabetes, is emphasized. It has been proven that stimulation of CB1 receptors leads to the stimulation of insulin, somatostatin, and glucagon secretion, while activation of CB2 receptors inhibits insulin secretion [28]. Many scientific studies highlight the influence of cannabinoid receptors and their agonists on the endocrine function of the pancreas [29,30,31].

The relationship between the endocannabinoid system and the development of insulin resistance and impaired glucose utilization has been confirmed in many research studies [28,29,30,31]. A study was conducted in mouse models to assess the function and regulation of the endocannabinoid system in fat cells and pancreatic beta cells. It was shown that blockade of the cannabinoid CB1 receptor reduced hyperinsulinemia, lipogenesis, and hypoadiponectinemia independently of weight loss, which directly translates into a reduced risk of developing obesity, insulin resistance, and type 2 diabetes [26]. Another study, also conducted on mouse models, aimed to confirm the theory that cannabinoid type 2 receptors play a key role in the pathogenesis of hyperlipidemia, obesity, and type 2 diabetes. Mice with induced diabetic cardiomyopathy were administered a natural cannabinoid, which is a selective agonist of the cannabinoid type 2 receptor. After administration of the phytocannabinoid, inhibition of oxidative stress and inflammation and a reduction in hyperglycemia were observed through activation of cannabinoid type 2 receptors [32]. Researchers Bermúdez-Silva et al. conducted studies in rats that assessed the role of the cannabinoid receptors CB1, CB2, and their agonists in the secretion of insulin, somatostatin, and glucagon [29,30,31]. It was observed that the activation of the CB2 cannabinoid receptors improves glucose tolerance, while blockade of these receptors leads to glucose intolerance. On the other hand, blockade of CB1 cannabinoid receptors produces an effect similar to the action of agonists of CB2 cannabinoid receptors [28,29].

It has been hypothesized that the occurrence of insulin resistance correlates with increased levels of endocannabinoids, particularly 2-arachidonoylglycerol, in adipose tissue; local increase in tissue 2-arachidonoylglycerol levels and subsequent CB1 receptor hyperactivity have been shown to closely correspond to decreased glucose metabolism in skeletal muscle, increased abdominal obesity, and increased lipid transport to the liver, which leads to the accumulation of free fatty acids in hepatocytes, which is associated with the development of insulin resistance [28]. Therefore, it can be concluded that the endocannabinoid system regulates glucose and lipid metabolism.

Endocannabinoids affect neural centers related to food intake, stimulate lipogenesis, and control human energy balance [33]. On the other hand, it has been observed that obesity from hyperalimentation negatively affects the secretion of insulin, leptin, and adiponectin [34]. Therefore, it can be concluded that the body’s energy balance is closely related to the presence of CB1 receptors. CB1 receptors are located in regions responsible for food intake, control of nutritional processes, and metabolism—elements of the hypothalamus (such as the lateral nucleus, paraventricular nucleus, and arcuate nucleus)—and peripherally, on neurons of the gastrointestinal tract. Their activation leads to the stimulation of appetite, which explains the increase in appetite after consuming cannabinoid substances [35].

Endocannabinoids regulate orexigenic pathways, thereby influencing the homeostatic balance that tends toward energy storage and weight gain. The clinically observed effect is increased appetite. It is hypothesized that the endocannabinoid system co-creates the orexigenic pathway, which is responsible for initiating food intake and reducing energy expenditure under conditions of hunger. Similar to the classical pathway controlled by neuropeptide Y, it is under negative control by leptin [36,37]. The endocannabinoid system participates in maintaining the body’s energy balance by stimulating lipogenesis and the production of metabolic reserves [17]. Endocannabinoids directly and indirectly influence increased glucose uptake and lipogenesis in adipose tissue and stimulate de novo synthesis of fatty acids and glucose in the liver [7]. Activation of the endocannabinoid system promotes obesity and obesity-related disorders such as glucose intolerance, type 2 diabetes, dyslipidemia, and metabolic syndrome, with subsequent increased risk of atherosclerosis and cardiovascular disease [7,18,19].

In summary, studies have confirmed the significant impact of endocannabinoids on glucose metabolism. It has been shown that their activation affects various metabolic functions, including insulin regulation, glucose secretion in the liver, and glucose homeostasis in muscles and adipose tissue [6,7,18,25,29]. Activation of CB1 receptors in the liver can lead to increased glucose secretion into the blood (gluconeogenesis) [38], which can contribute to elevated blood glucose levels (hyperglycemia), while activation of CB1 receptors in peripheral tissues (such as muscles and adipose tissue) can reduce tissue sensitivity to insulin, leading to the development of insulin resistance and significantly increasing the risk of type 2 diabetes [15,26,39]. Blocking CB1 receptors has been shown to improve tissue sensitivity to insulin and reduce blood glucose levels. Endocannabinoids can also affect insulin secretion from the pancreas. CB1 receptors in pancreatic cells are involved in regulating insulin secretion, which can influence blood glucose levels after a meal [37,40]. Pharmacological effects on cannabinoid CB1 and CB2 receptors of pancreatic β cells induce changes in insulin secretion, probably via regulation of intracellular calcium concentration [41,42]. Inhibition of CB1 receptors has been shown to have positive therapeutic effects. Induction of weight loss and reduction in obesity, improvement of glucose homeostasis and lipid profile, as well as reduction in fibrosis of parenchymal organs have been observed [40,43].

### 3.2. The Eating Disorders

Recently, the endocannabinoid system has been linked to metabolism, gut motility, and eating behaviors as well as chronic diseases such as obesity [37]. Research to date has provided information on the influence of the gut microbiome and diet on the endocannabinoid system [14,19,44]. Polyunsaturated fatty acids act on CB1 and CB2 receptors. Activation of CB1 receptors has been shown to lead to changes in eating habits that are partially dependent on leptin levels, while increased stimulation of CB1 receptors may lead to obesity [36,37]. Manipulation of the gut microbiome can also affect the endocannabinoid system. Studies conducted on animal models have shown increased mRNA expression of CB1 receptors in the small intestine, which subsided after fecal microbiota transplantation [44,45,46]. The actual studies refer to the connections between the endocannabinoid system and the immune system and nutritional status, partially mediated by the microbiome, in both health and disease. Modifying the endocannabinoid system through exercise, regulation of the gut microbiome, and consumption of ω-3 or ω-6 polyunsaturated fatty acids may provide new ways to treat nutrition-related diseases [44,47].

### 3.3. The Endocannabinoids in Chronic Liver Diseases

#### 3.3.1. Liver Fibrosis

Animal models have confirmed that inhibiting the CB1 receptor contributes to reducing liver fibrosis. Based on this, it has been hypothesized that CB1 receptor expression is increased in humans with liver cirrhosis and plays a key role in the pathogenesis of the disease [25,48]. In contrast, interaction with the CB2 receptor shows the opposite effect, as its activation induces antifibrogenic action in the liver and may play a protective role against liver fibrosis [45,49]. The function of both types of receptors is determined by their exact localization in the liver. The CB1 receptors are predominant in hepatocytes, stellate cells, and sinusoidal cells, while CB2 receptors are mainly found in stellate cells and Kupffer cells [48]. The activation of the liver endocannabinoid system in physiological condition is low because of the high expression of fatty acid amide hydrolase (FAAH) and monoacyloglycerol lipase (MAGL) responsible for ECS catabolism [50]. The upregulation of the endocannabinoids signaling pathway participates in transforming the hepatic stellate cells into myofibroblast cells and leads to portal hypertension [38,51].

#### 3.3.2. Liver Inflammation

The currently available literature confirms the pro- or anti-inflammatory effects of 2-arachidonoylglycerol, while anandamide mainly promotes anti-inflammatory effects [45,49]. Similarly, interactions with CB1 and CB2 receptors show opposing actions, acting in pro- and anti-inflammatory ways, respectively, in liver diseases [48]. Specifically, it has been shown that the CB1 receptor is involved in inflammation and the progression of steatohepatitis; consequently, the blockade of CB1 decreased oxidative stress in the liver and the production of proinflammatory cytokines [48,52]. The significant role in hepatic injury may mean bone marrow-derived macrophages expressed cannabinoid receptor 1, but the exact mechanism of this phenomenon is unknown [53,54]. During inflammatory and fibrotic processes in the liver, changes in activation of enzymes responsible for biosynthesis and degradation of endocannabinoids were observed. The activity of catabolic enzymes, e.g., FAAH, was remarkably decreased [53,55].

#### 3.3.3. Liver Cancer

The antitumor effect of anandamide has been documented in the case of cholangiocarcinoma, where it has been shown to inhibit tumor growth in intrahepatic cholangiocarcinoma (ICC) models in vitro and in vivo by activating the programmed cell death receptor complex [56]. However, Ma et al. showed that the other endocannabinoid 2-AG may promote tumorigenesis and metastasis in ICC [57]. The activity of diacyloglycerol lipase β (DAGLβ), the synthesizing enzyme of 2-AG, is upregulated and correlated with poor prognosis of patients with ICC [57]. In hepatocarcinogenesis, the activation of CB1 and CB2 receptors exerts opposite effects, similar to their role in fibrogenesis [58,59]. Lipidomic profiles can serve as novel biomarkers of early detection of hepatocellular carcinoma [49,60]. Influencing the endocannabinoid system at different levels opens up new therapeutic possibilities for liver diseases [61,62].

#### 3.3.4. Non-Alcoholic Fatty Liver Disease

Endocannabinoid signaling regulates energy homeostasis and is closely linked to non-alcoholic fatty liver disease (NAFLD) [50]. Dysregulation of the ECS, particularly overactivation of CB1 receptors, contributes to hepatic steatosis, insulin resistance, and progression to non-alcoholic steatohepatitis. CB1 activation increases de novo lipogenesis and fat accumulation in the liver. The intensity of lipolysis is decreased. The activation of CB2 receptors may counteract some harmful effects [38,63]. It was shown that serum endocannabinoid ligands, namely anandamide (AEA) and 2-arachidonoylglycerol (2-AG), are significantly higher in patients with NAFLD compared with healthy individuals [64,65,66]. A study was conducted to elucidate the role of the endocannabinoid system in the treatment of NAFLD using docosahexaenoic acid (DHA) [64]. Based on targeted ceramide analysis and lipidomic studies [64,65,66], it was confirmed that inhibiting endocannabinoid signaling resulted in reduced de novo fatty acid synthesis and increased expression levels of proteins associated with β-oxidation of fatty acids. The study provided evidence that docosahexaenoic acid supplementation affects non-alcoholic fatty liver disease by suppressing the endocannabinoid system [64].

### 3.4. The Endocannabinoid System in Gastrointestinal Disorders

The endocannabinoids affect the digestive system by immune and sensory function. They regulate motility, visceral sensation, mucosal integrity, gut–brain interactions, and inflammatory mechanisms through CB2 receptors, and indirectly through neurotransmitters such as gamma-aminobutyric acid (GABA) and glutamate [67,68]. It is postulated that the anti-inflammatory effects of cannabinoids in gastrointestinal disorders occur by reducing inflammatory factors like myeloperoxidase activity and regulating cytokine levels [46]. Current research is focused on the role of phytocannabinoids in gastrointestinal functions and the potential therapeutic applications of these compounds [69,70]. There is a prospect of using cannabinoids as elements of therapy for gastrointestinal disorders such as Crohn’s disease, ulcerative colitis, or irritable bowel syndrome to reduce abdominal pain and inflammation [46]. They relieve symptoms connected with gastroparesis and diverse nausea [69,70]. Endocannabinoids are also seen as one of the factors influencing the gut microbiome [71].

### 3.5. Endocannabinoids in Neuropsychiatric Disorders

#### 3.5.1. Stress-Related

Dysregulation of the endocannabinoid system is associated with the development of stress-related neuropsychiatric diseases [72,73]. A meta-analysis assessed the response of endocannabinoids to acute psychosocial stress in individuals and compared the results with the control group. It was shown that baseline levels of AEA and 2-AG were increased in individuals with stress compared with the control group [72]. As is known, stress induces excitatory responses in limbic areas of the brain that are important for emotions [73]. In response to excessive stress, there is an increased secretion of 2-arachydonylglycerol, which leads to desensitization of the CB1 receptor. This confirms the protective role of stress adaptation and anxiety control [74,75].

Mental disorders are one of the most common categories of disease burden worldwide. It is predicted that by 2030, depression will be the most common cause of global mental health burden [76]. The COVID-19 pandemic has probably contributed to the exacerbation of these symptoms, especially among young people who have experienced isolation, disruption of daily activities, and limited access to healthcare [72,76]. Recently, a large role has been attributed to the endocannabinoid system in the pathophysiology of depression and anxiety [74]. In particular, cannabinoids capable of selectively interacting with the cannabinoid receptor type 2, exhibiting immunomodulatory and anti-inflammatory properties, seem to be excellent therapeutic agents in the treatment of neuropsychiatric disorders with limited or absent psychotropic activity [75,77,78].

It is not fully understood whether the therapeutic effects of CB2 receptor ligands are due to the improvement of inflammation via modulation of CB2 receptor activity or via other signaling pathways in the endocannabinoid system [72,77]. Further studies are needed to assess the role of the CB2 receptor in neuropsychiatric disorders [77].

The endocannabinoid system is present in the peripheral nervous system. A study was conducted to assess its neuroprotective role [74,78]. It was suggested that activation of the cannabinoid CB1 receptor may contribute to the restoration of homeostasis in various neuropathological and neuropsychiatric conditions associated with dysregulation of the hypothalamic–pituitary–adrenal axis [23,78].

#### 3.5.2. Related to Autism Spectrum

Recent studies have shown a significant involvement of the endocannabinoid system in the pathogenesis of neuropsychiatric disorders, including autism spectrum disorders (ASDs) [79,80,81,82]. Autism spectrum disorders are characterized by difficulties in social communication as well as repetitive behaviors, interests, or activities, often accompanied by cognitive limitations.

Available studies suggest that a probable cause of the disease may be an imbalance in the endocannabinoid system [79,80,81,82]. This confirms the significant impact of endocannabinoids on the functioning of the nervous system and cognitive development. Evidence presented in mouse models suggests that enhancing anandamide signaling by inhibiting its breakdown promotes prosocial behaviors [83]. Additionally, it has been shown that acute or chronic inhibition of CB1 receptors has a positive effect on cognitive functions [83]. Nevertheless, autism spectrum disorders are complex neurodevelopmental disorders, and the role of the endocannabinoid system in the pathogenesis of this disease seems undeniable, although further research is needed to fully understand the complex interactions between parallel systems that regulate late brain development [84].

#### 3.5.3. Parkinson’s Disease

Numerous studies confirm the role of endocannabinoids in controlling the functions of dopaminergic neurons [85,86,87]. Recently, the mutual regulation of endocannabinoid–dopamine neurotransmission has been closely examined, with particular emphasis on the actions of endocannabinoids in ion and synaptic signaling in dopaminergic neurons mediated by cannabinoid receptors or others [85,88]. Reduced endocannabinoid system activity has been documented in mouse models of Parkinson’s disease. Fatty acid amide hydrolase (FAAH) inhibitors and cannabinoid CB1 receptor antagonists have also been shown to alleviate Parkinson’s symptoms [86]. Increased expression of the cannabinoid CB1 receptor in the basal ganglia has been noted in mouse models of Parkinson’s disease. This represents a pathological process that contributes to disease progression or a compensatory mechanism for damaged dopaminergic neurons in the substantia nigra [87,88]. A full understanding of the mutual interactions between endocannabinoids and dopamine may provide new therapeutic strategies for managing pain in Parkinson’s disease in the future.

#### 3.5.4. Alzheimer’s Disease

The endocannabinoid system is a well-studied system that influences various physiological functions, plays a significant role in many metabolic activities, and has certain neuroprotective properties [81,86,88,89]. In Alzheimer’s disease, activation of the endocannabinoid system may provide neuroprotection by regulating certain neuronal pathways through complex molecular cascades [88,89]. Numerous studies are being conducted on the modulators of cannabinoid receptors (CB1 and CB2) and cannabinoid enzymes in Alzheimer’s disease [89,90,91]. In particular, the modulation of CB1 or CB2 receptors reduces the concentration of proinflammatory cytokines such as IL-2 and IL-6 and reduces the activation of microglia, which contributes to the inhibition of the inflammatory response in neurons [89,91]. Additionally, naturally occurring cannabinoid metabolic enzymes may provide significant neuroprotection. The multidirectional neuroprotective properties of phytocannabinoids and their regulatory capabilities affecting the endocannabinoid system could provide significant benefits in mitigating Alzheimer’s disease [90]. In the study by Ramarez et al. using rat models, activation of the cannabinoid CB1 receptor was shown to prevent β-amyloid-induced neurotoxicity [89]. Moreover, increasing the expression of the CB1 receptor contributes to the reduction in Alzheimer’s disease symptoms such as cognitive impairment and memory deficits. Inhibition of fatty acid amide hydrolase (FAAH), which degrades endogenous cannabinoids, may also be of great importance in the treatment of Alzheimer’s disease [86,91].

### 3.6. The Endocannabinoid System and Pain Perception

The mechanisms of severe abdominal pain, which occurs in acute pancreatitis, are still poorly understood. The endocannabinoid system is thought to play a role in pain perception in this disease [92]. Studies have shown that CB2 receptor agonists reduce the pain perception in acute pancreatitis, while the effect of CB1 receptor agonists on pain severity depends on the progression of the disease [92,93,94]. However, the exact processes by which CB receptors influence pain in acute pancreatitis remain unclear and require further research [80].

Recently, there have been increasing reports aimed at understanding the therapeutic potential of endocannabinoids in treating neuropathic pain [92,93,94,95,96]. Advances in endocannabinoid research have shown significant potential in treating chronic neuropathic pain [92,94,96]. Currently, key research areas include the localization of CB1 and CB2 receptors and their role in pain modulation in the central nervous system [93,94]. Quintero et al. showed that the therapeutic effects of endocannabinoids can be enhanced by regulating their endogenous levels, particularly by inhibiting their metabolism by enzymes [93]. Dysregulation of the endocannabinoid system, microglial activation, and interactions between various signaling pathways contribute to the onset and maintenance of neuropathic pain [93]. Understanding these molecular and cellular processes is crucial for developing targeted therapies that utilize the endocannabinoid system to alleviate neuropathic pain.

The endocannabinoid system has been documented to be involved in the regulation of nociception [94,95]. The involvement of CB1 and CB2 receptors in pain regulation has been confirmed. The role of endocannabinoids in the control of neuropathic and inflammatory pain is extensively discussed in the review article by Donvito et al. [96].

### 3.7. Endocannabinoids in Skeletal Disorders

A study was conducted to assess the immunomodulatory role of the endocannabinoid system in intervertebral disc degeneration [97]. The study involved 20 healthy volunteers (control group) and 40 patients with intervertebral disc degeneration (disease group). It was showed that the signaling molecules of the endocannabinoid system, 2-arachidonoylglycerol and anandamide, were significantly lower in diseased discs compared with the control group. The study suggests that endocannabinoids may contribute to susceptibility to infections and inflammation within intervertebral discs, which could translate into a potential therapeutic target for improving disc health [97].

Another study of 23 patients assessed changes in endocannabinoid levels before and after knee or hip replacement surgery [98]. A significant decrease in all endocannabinoids was found after surgery. Furthermore, no differences were observed between knee and hip replacement surgery or between sexes. The results of the study suggest that the endocannabinoid system may be a pharmacological target in orthopedic surgery [95,98].

### 3.8. The Role of Endocannabinoids in the Muscular System

A review focusing on two endocannabinoids, 2-arachidonoylglycerol and anandamide, highlighted their specific actions in skeletal muscles [99]. Particular attention was paid to the influence of endocannabinoids on the contractile activity of muscle fibers, the secretion of transmitters in motor synapses, and energy exchange [99,100]. Myoendocannabinoids appear to play a significant role in increasing overall endocannabinoid levels in the blood during muscle exercise and the occurrence of the so-called “runner’s high”, and they likely play an important role in correcting various psychophysiological conditions such as pain syndromes or stress [12,99].

A feature of cancer cachexia is muscle degeneration. The endocannabinoid system, primarily cannabinoid receptor 1, regulates muscle processes including metabolism, anabolism, and regenerative capacity [100,101]. Based on an animal model study, it has been documented that CB1 receptor expression correlated with muscle mass, metabolism, and catabolism markers [101]. This indicates the growing role of the endocannabinoid system in muscle physiology.

### 3.9. Endocannabinoids and the Development of Cardiometabolic Diseases

A study involving 133 young adults aimed to demonstrate the relationship between plasma levels of endocannabinoids and their analogs with body composition and cardiometabolic risk factors [102]. It was observed that plasma levels of endocannabinoids and their analogs were higher in overweight and obese individuals compared with metabolically healthy study participants. Higher plasma levels of endocannabinoids and analogs correlate with a poorer metabolic profile in young adults and are associated with the development of cardiovascular diseases [102,103,104].

#### Myocardial Infarction

Recent reports suggest that the endocannabinoid system is associated with diseases affecting the cardiovascular system, such as myocardial infarction [102,103,104,105]. The CB2 receptor is expressed in cardiomyocytes and plays a key role in alleviating pathological changes in the heart muscle associated with myocardial infarction [105]. Activation of CB2 receptors has a cardioprotective effect in myocardial infarction through numerous molecular pathways, protecting cardiomyocytes from ischemic damage [103,104,106]. The molecular correlation of CB2 receptors with heart damage markers such as troponin I, LDH1, and CK-MB is particularly studied [105]. In an experimental model of myocardial infarction, treatment with a CB2 receptor agonist balanced hemodynamic parameters and reduced levels of heart damage markers such as CK-MB, LDH, and troponin, suggesting a cardioprotective role of the CB2 receptor [105]. Activation of the CB2 receptor in vascular endothelial cells reduces TNF-α-induced production of VCAM-1 and ICAM-1, thereby reducing cardiovascular risk [105]. Pharmacological actions based on the regulation of CB2 receptors may find applications in ischemic heart disease, including myocardial infarction.

Increased expression of cannabinoid receptor type 1 is associated with the occurrence of atherosclerosis and leads to the formation of atherosclerotic plaques [103]. Coronary atherosclerosis is one of the causes of coronary artery disease. The risk of myocardial infarction increases significantly with the progression of myocardial hypertrophy [106]. It has been proven that reduced expression of the CB2 receptor is associated with myocardial hypertrophy, while inhibition of the CB1 receptor slows down hypertrophic changes in the heart [103,104,106]. In addition, activation of CB2 receptors delays myocardial fibrosis caused by infarction, which significantly reduces the risk of arrhythmia and improves heart function [104,106].

### 3.10. Endocannabinoids and the Reproductive System

The endocannabinoid system plays a key role in fertilization and implantation, regulates placental function, and participates in childbirth [107,108]. A properly functioning endocannabinoid system is essential for maintaining physiological pregnancy from the moment of embryo implantation until delivery [107]. Close cooperation of all components of the endocannabinoid system ensures the proper functioning of reproductive organs [107]. Endocannabinoids activate the CB1 receptor and induce the production of prostaglandins, which cause uterine contractions, and thus, childbirth [108]. Currently, new predictors of preterm birth in the endocannabinoid system are being sought. Preterm birth is one of the greatest challenges in obstetrics; despite advances in medicine, there are no sufficiently effective methods for its diagnosis, treatment, and prevention. Children born prematurely die more often, fall ill, are more often hospitalized, and achieve poorer educational results, and the risk of these adverse events increases with decreasing gestational age at delivery [107,108]. Assessment of endocannabinoid concentrations in the blood of pregnant women may increase the sensitivity of predicting the occurrence of preterm birth [108,109]. The study by Parižek et al. assessed the levels of AEA and other endocannabinoids such as 2-arachidonoylglycerol (2-AG), 2-linoleoylglycerol (2-LG), 2-oleoylglycerol (2-OG), and 2-arachidonoyldopamine (NADA) in the blood of pregnant women. It was shown that at least two endocannabinoids, AEA and 2-AG, can be considered predictors of preterm birth [109]. At present, further research is needed to ultimately confirm the role of endocannabinoid measurement in detecting preterm birth.

#### Endometriosis

Endometriosis is a disease of the female reproductive system characterized by the presence of cells from the lining of the uterus (endometrium) outside the uterine cavity. Endometriosis is a cause of infertility, chronic pain, and a resulting deterioration in the quality of life in many women. The disease most often affects women of reproductive age. Its exact cause has not yet been determined [110].

It is known that the endocannabinoid system affects several cardinal features of this complex disease, including pain, vascularization, and lesion formation, but the exact mechanisms are unknown [110,111]. The reduced expression of the CB1 receptor seems to be associated with the development of endometriosis, and its regulation may provide anti-inflammatory effects [110,111]. The endocannabinoid system is involved in numerous processes related to the disease’s progression, such as cell proliferation, apoptosis, cell migration, inflammation, and interaction with sex hormones [110].

Using mouse models, researchers have attempted to elucidate the role of the endocannabinoid system in the initiation, progression, and immunological modulation of the disease [111]. The study provided evidence of the involvement of an imbalance between CB1 and CB2 receptors in the pathogenesis of endometriosis and forms the basis for developing targeted therapies [111].

### 3.11. Endocannabinoids and Eosinophilic Asthma

The CB2 receptor may contribute to the pathogenesis of eosinophilic asthma. Current research results provide new insights into the molecular signaling mechanism in this disease [112,113]. One of the endocannabinoids, oleoylethanolamide (OEA), and the expression of its cognate CB2 receptor, was significantly increased in eosinophilic asthma [112]. OEA caused the activation of eosinophils by the CD69 molecule and infiltration of the airways along with increased production of proinflammatory cytokines in bronchoalveolar fluid [113]. Frei et al. examined human peripheral blood eosinophils from symptomatic allergic donors and mouse bone marrow-derived eosinophils [114]. The CB2 receptor expression, the ability to adhere, chemotaxis, and the production of reactive oxygen species were significantly higher in allergic human blood eosinophils. The endocannabinoid 2-AG was proposed to elicit eosinophil activation [114]. This showed the contribution of the CB2 receptor to eosinophil-driven diseases. In metabolomics and genetics study, the endocannabinoid linoleoyl ethanolamide was identified as a novel genetically driven metabolite with asthma associations [115]. The level of the most well-known endocannabinoid, anandamide, increases in bronchoalveolar fluid after exposure to allergens in allergic asthma and increases human airway epithelial cell permeability due to arachidonic acid metabolites [116,117].

In addition, a proinflammatory role of 2-AG has been demonstrated that is a chemotactic factor for eosinophilia, and this reaction is regulated by the CB2 receptor [115,118]. 2-AG, acting on endothelial cells, can promote leukocyte recruitment and transmigration [118]. Studies on mouse models indicate that in allergic bronchitis, the concentration of 2-AG is significantly increased, which strongly correlates with the infiltration of immune cells and the severity of the disease [119,120]. Blocking the CB2 receptor prevents the migration of inflammatory cells [115,118,119,120].

### 3.12. Endocannabinoids in Skin Disorders

Maintaining a healthy skin barrier largely depends on the structure and composition of the lipid layers in the stratum corneum [121,122]. Furthermore, it has been shown that the endocannabinoid system, which regulates skin cell proliferation, differentiation, and survival, plays a major role in maintaining an effective dermal–epidermal barrier [121,122,123,124]. A study was conducted in which it was shown that moisturizers containing physiological lipids and functional ingredients (e.g., endocannabinoids) are beneficial in treating disorders associated with impaired dermal–epidermal barrier function [121].

A study by Ständer et al. determined the exact location of CB1 and CB2 receptors in the skin [122]. Their presence was documented in skin nerve fibers, macrophages, mast cells, keratinocytes, sebocytes, hair follicle cells, and sweat glands [122]. The endocannabinoid system is involved in skin homeostasis, and its dysregulation contributes to the development of many dermatological diseases [121,122,123]. Numerous studies have shown a relationship between endocannabinoid CB1 or CB2 receptors and allergic contact dermatitis, atopic dermatitis, pruritus, psoriasis, acne, and seborrheic dermatitis [122,123,125]. A study conducted on mouse models observed a relationship between the endocannabinoid system and hair growth [125]. A synthetic CB1 receptor antagonist stimulated hair growth in mice. The data obtained indicate that CB1 receptor antagonists can prevent hair loss [125]. Strong evidence also suggests an anti-aging role for the CB1 receptor [124]. Mice with a genetic deletion of the CB1 receptor showed histological signs of skin aging, including reduced subcutaneous adipose tissue, reduced collagen production, and increased production of proinflammatory cytokines [124].

### 3.13. The Endocannabinoid System in Kidney Diseases

#### 3.13.1. Kidney Disease Associated with Obesity and Metabolic Syndrome

The benefits of CB1 receptor blockade have been confirmed in the kidneys of obese insulin-resistant rats, where treatment with rimonabant (a selective CB1 receptor antagonist) prevented kidney function loss and reduced glomerular fibrosis with concurrent normalization of body weight, fasting glucose levels, and lipid parameters [52]. Other studies have provided evidence of the direct impact of the endocannabinoid system on the kidneys [126,127,128,129]. Inhibition of the CB1 receptor and/or activation of the CB2 receptor positively affects the lost balance between these pathways, translating into improved kidney function [52,127].

Elevated endocannabinoid levels observed in both obese animals and humans suggest a potential link with chronic kidney disease induced by obesity [52,130]. A study by Permyakova et al. assessed the biochemical blood analysis, kidney tissue histology, and endocannabinoid level in a group of 21 lean and obese men [130]. In the obese group, increased parameters of azotemia and fibrosis were observed, which indicated kidney damage. Serum endocannabinoid levels were similar in the lean and obese groups. However, renal anandamide levels were higher in the obese patients [130]. Obese individuals also showed reduced expression of cannabinoid receptor 1 in the kidneys along with increased activity of enzymes that synthesize and degrade endocannabinoids. The study provided evidence of the relationship between the endocannabinoid system in the kidneys, kidney damage markers, obesity, and related pathologies [130].

#### 3.13.2. Diabetic Kidney Disease

The endocannabinoid system plays a role in the pathogenesis of diabetic nephropathy. Numerous studies have shown that in diabetic kidney disease, there is an imbalance in the endocannabinoid system characterized by harmful CB1 receptor expression outweighing the protective CB2 receptor expression [131,132,133,134]. Animal studies have confirmed that restoring the balance between CB1 and CB2 receptors reduces albuminuria and prevents the loss of nephrin and podocin, which are key components of the filtration membrane [132,134]. Regulation of the endocannabinoid system is possible with selective agonists that cause peripheral blockade of the CB1 receptor and activation of the CB2 receptor [132]. Results of other studies have suggested that inhibition of the CB1 receptor or activation of the CB2 receptor may reduce the changes in the body caused by diabetes [133,134]. Animal studies have analyzed the effect of a CB2 agonist administered during the active phase of the disease on functional and structural kidney changes in type 2 diabetic nephropathy [132,134]. These studies confirmed the renoprotective effect of the CB2 agonist, which was similar to the effect of an ACE inhibitor. This represents a promising therapeutic option for the potential treatment of diabetic nephropathy [132,134]. Not only glomerulopathy, but also tubulopathy is involved in the pathogenesis of diabetic kidney disease. The ECS due to CB1 receptor regulates the expression, translocation, and activity of the glucose transporters (GLUT2) located in the proximal tubules [135].

#### 3.13.3. Non-Diabetic Chronic Kidney Diseases

Recent data indicate the role of endocannabinoids not only in diabetic kidney disease but also in chronic kidney disease unrelated to diabetes [127,128,129]. In many nephropathies, increased renal CB1 receptor expression is observed, primarily in myofibroblasts, which correlates with kidney function and leads to fibrosis [136,137]. The renin–angiotensin–aldosterone system (RAS) plays an important role in the physiological regulation of volemia, water and electrolyte balance, and blood pressure. Individual components of this system may also participate in the pathogenesis of hypertension and its cardiovascular complications as well as in the pathogenesis of chronic kidney disease [128]. Interaction between the RAS and the endocannabinoid system has been demonstrated in the kidneys [128]. Activation of the CB1 receptor may interact with the angiotensin type 1 (AT1) receptor, activating it and contributing to the harmful effects of angiotensin II on the body [128]. Chronic kidney disease affects millions of people worldwide, and so far, there are few therapeutic strategies available. The endocannabinoid system seems to be of great importance in light of the potential therapeutic applications in the prevention of chronic kidney disease. The most important receptor in the kidneys is the CB1 receptor, and its endogenous local ligands are anandamide and 2-arachidonoylglycerol [138].

In a healthy kidney, the CB1 receptor is expressed in many cell types, especially in the vascular system, where it is involved in regulating renal hemodynamics [139]. Additionally, the CB1 receptor may participate in water–sodium balance and blood pressure regulation, but its exact role remains unclear [139,140]. Current knowledge allows us to unequivocally state that increased CB1 receptor expression promotes kidney fibrosis in both metabolic and non-metabolic nephropathies [141,142]. In metabolic syndrome, obesity, and diabetes, CB1 receptor inhibition not only improves metabolic parameters but also plays a direct role in preventing kidney fibrosis [142]. It has also been confirmed that CB1 receptor expression was significantly increased in kidney biopsies of patients with IgA nephropathy and acute interstitial nephritis [136,142].

#### 3.13.4. Kidney Transplantation

A study on the expression of the CB1 receptor in transplanted kidneys has also been performed [140]. It has been shown that CB1 receptor expression significantly increases within the first three months after kidney transplantation, and then remains stable. It has been hypothesized that the high level of CB1 receptor expression in transplanted kidney biopsies may be a consequence of acute tubular necrosis caused by cold ischemia and may therefore correlate with the estimation of further kidney fibrosis development. Additionally, patients with stable kidney fibrosis during the first year after transplantation tended to have a smaller increase in CB1 receptor expression than patients with progressive kidney fibrosis [140]. Therefore, assessing CB1 receptor expression may be related to the early development of chronic kidney disease or at least be a marker of kidney fibrosis [140].

The population of people in advanced stages of chronic kidney disease and those with end-stage renal disease requiring hemodialysis is steadily growing [143,144]. Mortality in patients with end-stage renal failure undergoing hemodialysis remains high. Cachexia in hemodialysis patients is a risk factor for poor prognosis. While obesity and higher BMI are associated with an increased risk of death in the general population, in some patient populations, including those with chronic obstructive pulmonary disease, acquired immunodeficiency syndrome, or chronic heart failure, the opposite trend has been observed. This phenomenon, known as the “obesity paradox”, has also been well documented in patients with end-stage renal failure, where higher BMI is associated with a reduced risk of cachexia and death [128,145].

A study was conducted on a group of hemodialysis patients measuring serum levels of the main ligands of the endocannabinoid system [143]. The results were correlated with various clinical and laboratory indicators and observed for their association with mortality over the next 12 months. The results showed that increased serum levels of 2-arachidonoylglycerol positively correlated with higher serum triglyceride levels, lower serum high-density lipoprotein (HDL) levels, and higher body mass indices measured by anthropometric measurements [143]. Additionally, it was shown that higher serum levels of 2-arachidonoylglycerol were associated with a reduced risk of death in hemodialysis patients [143]. It was observed that serum anandamide levels did not show the same correlation as 2-arachidonoylglycerol, and in some analyses, it was found that the two endocannabinoids had opposing effects [126,127]. These findings are intriguing, considering that both anandamide and 2-arachidonoylglycerol are considered key ligands of the endocannabinoid system. Further research is needed to determine the role of the endocannabinoid system in end-stage renal failure.

#### 3.13.5. Appetite in Hemodialysis Patients

Uremia-related malnutrition may be associated with fluctuations in circulating endocannabinoid levels and similar compounds known to affect appetite [128]. A study was conducted to demonstrate the relationship between end-stage renal disease and the presence of endocannabinoids and other appetite-related molecules [144]. Endocannabinoid-like compounds were identified that were significantly associated with appetite and satiety in patients undergoing chronic hemodialysis. The study was conducted on hemodialysis patients and a control group not undergoing hemodialysis [144]. Appetite was assessed using the Simplified Nutritional Appetite Questionnaire (SNAQ). Strong correlations were observed between specific endocannabinoid-like compounds and SNAQ questionnaire results in study participants. These findings confirm the relationship between circulating endocannabinoids and appetite in hemodialysis patients. Further research is needed to determine whether supplementation with endocannabinoid-like substances can improve appetite in patients with uremic malnutrition [144]. A recent study showed that the gut microbiome modulates biologically active metabolite levels, including endocannabinoid-like mediators, and leads to cognitive alterations in CKD patients [32].

#### 3.13.6. Acute Kidney Injury

There is evidence that the endocannabinoid system plays a role in acute kidney injury such as drug-induced nephrotoxicity caused by nephrotoxic substances like cisplatin or prerenal acute kidney injury from hypoperfusion [129,133].

Cisplatin is an important chemotherapeutic agent, but its nephrotoxicity limits its clinical use. Studies on cisplatin-induced nephrotoxicity have shown no changes in CB1 receptor expression, but anandamide levels in the kidneys were elevated [129,133,134]. It was also shown that both CB1 receptor blockade and CB2 receptor activation reduce cisplatin-induced inflammatory response, oxidative stress, and cell death in the kidneys and improve kidney function, while CB2 receptor inhibition exacerbates inflammation and tissue damage. This is likely due to oxidative stress and inflammation, as both CB1 receptor blockade and CB2 receptor activation reduce the expression of NADPH oxidases (NOX2, NOX4) and proinflammatory cytokines (TNF-α, IL-1β) [128,129,145]. Thus, the endocannabinoid system, through CB2 receptors, protects against cisplatin-induced kidney injury by reducing inflammation and oxidative stress. Selective CB2 receptor agonists may represent a promising new approach to preventing this debilitating chemotherapy complication [145].

Currently available data on the role of the endocannabinoid system in prerenal hypoperfusion-induced kidney injury are limited. In this mechanism, both CB1 and CB2 receptor activation prevented hypoperfusion-induced kidney injury [126,141]. In a study conducted by H. Moradi et al. at a center in California, the role of the endocannabinoid system in prerenal hypoperfusion-induced kidney injury was observed in mouse models [146]. This study was the first to document the association between increased renal 2-arachidonoylglycerol levels and improved kidney function in hypoperfusion-induced acute kidney injury. Pharmacological regulation of 2-arachidonoylglycerol aimed at CB2 receptor activation showed improved kidney function despite a slight increase in inflammation [146]. Further research is needed to determine the mechanisms responsible for the observed effects and the potential therapeutic value of 2-arachidonoylglycerol in prerenal hypoperfusion-induced kidney injury.

#### 3.13.7. Autosomal Dominant Polycystic Kidney Disease

Autosomal dominant polycystic kidney disease (ADPKD) is a commonly inherited disorder characterized by the formation of kidney cysts [147]. ADPKD leads to progressive kidney damage, and metabolic changes, mainly related to glucose metabolism, are commonly observed in patients with this condition. Many individuals with ADPKD may experience insulin resistance and problems controlling blood glucose levels, which are associated with general inflammation, kidney dysfunction, and obesity. The main pathological feature of ADPKD is the development of interstitial inflammation.

In the context of ADPKD, the endocannabinoid system may play a role in both glucose homeostasis regulation and the inflammatory response that contributes to disease progression [126,127]. One of the main mechanisms of kidney damage in ADPKD is chronic inflammation, which contributes to the development and progression of the disease [147]. Endocannabinoids, especially CB1 receptor activation, can exacerbate kidney inflammation, worsening the disease course. Conversely, CB2 receptors have anti-inflammatory effects, and their activation may limit tissue damage, including in the kidneys [127,128,129]. Many individuals with ADPKD experience insulin resistance, which is associated with the development of type 2 diabetes. The endocannabinoid system, particularly in the context of CB1 receptors, can exacerbate insulin resistance and glucose metabolism disorders [28,29,30,31]. There is a link between obesity and CB1 receptor activation, and obesity is a common problem in patients with ADPKD, which can further worsen glucose metabolism [148].

In a study by J. Klawitter at the University of Denver, the relationship between the endocannabinoid system and ADPKD was observed [148]. The study included a group of 102 patients with ADPKD and a control group of 100 healthy volunteers. Compared with healthy individuals, patients with ADPKD had higher levels of interleukin-6 and -1b as well as lower levels of anandamide, 2-arachidonoylglycerol, and their plasma compounds. The study results suggest that patients with ADPKD have lower levels of endocannabinoids [148]. Restoring the balance of the endocannabinoid system in the kidneys, for example, by increasing the levels of anandamide, 2-arachidonoylglycerol, or the anandamide analog palmitoylethanolamide (PEA) through increased synthesis or reduced degradation, may be beneficial and could represent a promising therapeutic target for patients with this progressive disease. Despite the importance of the endocannabinoid system in kidney physiology, this study was the first to show changes in the endocannabinoid system in patients with ADPKD [148].

## 4. Conclusions

The endocannabinoid system plays a crucial role in maintaining physiological balance and regulating functions such as pain perception, immune response, metabolism, and neurological processes [6,7]. Due to the multifaceted biological actions of the components of the ECS, researchers are seeking agonists/antagonists of cannabinoid receptors or other kinds of compounds with potential applications in targeted pharmacotherapy aimed at the endocannabinoid system. Although plant-derived cannabinoids have long been used in medicine, there are increasing attempts to use synthetic compounds as ligands for cannabinoid receptors or modulators of enzymes involved in endocannabinoid metabolism. Rimonabant, a selective CB1 receptor antagonist, was registered in Europe for the treatment of obesity from 2006 to 2008, particularly in patients with type 2 diabetes or metabolic syndrome [52]. However, the European Medicines Agency determined that the risks of using the drug outweighed its benefits due to the serious psychiatric side effects, including depression and suicidal thoughts. Several novel drugs targeting ECS are under investigation [149]. Notable examples include endocannabinoid reuptake inhibitors (eCBRIs) such as SYT-510 (currently under development), which are designed to treat anxiety, mood, and traumatic stress disorders, and AM404, an active metabolite of paracetamol, which inhibits AEA uptake. The other group consists of inhibitors of enzymes degrading AEA (fatty acid amide hydrolase—FAAH) and 2-AG (monoacyloglycerol lipase—MAGL) being explored for their potential in treating anxiety and pain [149]. Drugs that modulate ECS hold promise for a variety of therapeutic applications, including glucose metabolism, obesity, neuroprotection, psychiatric disorders, pain management, and inflammation control, also in the context of chronic diseases. Further studies are needed to fully understand the complexities of this system and develop safe, effective treatments.

## Data Availability

Data sharing not applicable. No new data were created or analyzed in this study.

## Supplementary Materials

The following supporting information can be downloaded at: https://www.mdpi.com/article/10.3390/jcm14082851/s1, Table S1. Studies evaluating endocannabinoids’ role in health and disease.
