# Supplementary material for: The Role of Endocannabinoids in Physiological Processes and Disease Pathology: A Comprehensive Review
Source: J Clin Med. 2025 Apr 21;14(8):2851. doi: 10.3390/jcm14082851 (PMC12027566; doi:10.3390/jcm14082851)
Supplement: Supplementary file 1 [file jcm-14-02851-s001.zip › jcm-3507608-supplementary.pdf]

Table S1. Studies evaluating endocannabinoids role in health and disease.

AUTHOR	TITLE OF THE WORK	YEAR	STUDY SUBJECT/FINDINGS	CLINICAL STUDY / REVIEW
M.Biernacki, E.Skrzydlewska	Metabolism of endocannabinoids	2016	Mechanisms of synthesis and degradation of AEA and 2-AG	review
G. Kunos, D. Osei-Hyiaman, J. Liu, G.Godlewski, S. Bátkai	Endocannabinoids and the Control of Energy Homeostasis	2008	The role of the endocannabinoid system (EKAN) in regulating energy homeostasis	review
F. J. Bermúdez-Silva, J. Suárez Pérez, A.Nadal, F.Rodríguez de Fonseca	The role of the pancreatic endocannabinoid system in glucose metabolism	2009	Presence of the endocannabinoid system (EKAN) in the pancreatic islets of Langerhans; the role of endocannabinoids in hormone secretion and glucose homeostasis	review
M. Borowska, A. Czarnywojtek, N.Sawicka-Gutaj, K. Woliński, M. T. Płazińska, P. Ł. Mikołajczak, et al.	The effects of cannabinoids on the endocrine system	2019	Assessment of the impact of the endocannabinoid system (EKAN) on endocrine system function, energy balance, and glucose homeostasis	review
G. Dörnyei, Z. Vass, C. B. Juhász, G. L. Nádasy, L. Hunyady, M. Szekeres	Role of the Endocannabinoid System in Metabolic Control Processes and in the Pathogenesis of Metabolic Syndrome: An Update	2023	The role of the endocannabinoid system (EKAN) and CB1 receptor signaling in regulating energy and metabolic homeostasis, in the development of obesity and metabolic syndrome	review
L. C. Gowatch, J.M. Evanski, S.L. Ely, C. G Zundel, A.Bhogal, C. Carpenter, et al.	Endocannabinoids and Stress-Related Neurospsychiatric Disorders: A Systematic Review and Meta-Analysis of Basal Concentrations and Response to Acute Psychosocial Stress	2024	Assessment of endocannabinoid levels in neuropsychiatric disorders related to stress	review
C.Ricardi, S.Barachini, G.Consoli, D. Marazziti, B. Polini, G. Chiellini	Beta-Caryophyllene, a Cannabinoid Receptor Type 2 Selective Agonist, in Emotional and Cognitive Disorders	2024	Assessment of the anti-inflammatory and immunomodulatory properties of BCP (CB2 receptor ligand) in the treatment of depression and anxiety	review

O. P Balezina, E. O Tarasova, P. O Bogacheva	Myogenic Classical Endocannabinoids, Their Targets and Activity	2024	Assessment of the effects of 2-AG and AEA in skeletal muscles	review
S. Dalle, C. Hiroux, K. Koppo	Endocannabinoid remodeling in murine cachexic muscle associates with catabolic and metabolic regulation	2024	Assessment of the impact of cachexia and physical exercise on endocannabinoid signaling in muscles. Evaluation of the correlation between CB1 receptor expression and markers of muscle anabolism, catabolism, and metabolism.	Clinical study
L.Russell, K. Condo, T. DeFlorville	Nutrition, endocannabinoids, and the use of cannabis: An overview for the nutrition clinician	2024	Assessment of the impact of endocannabinoids on metabolism and nutrition	review
H. Lingegowda, K. B Zutautas, Y. Wei, P. Yolmo, D. J Sisnett, A. McCallion, et al.	Endocannabinoids and their receptors modulate endometriosis pathogenesis and immune response	2024	The role of the endocannabinoid system in the pathogenesis of endometriosis	Clinical study on mouse models.
M.Mancini, A.Calculli, D. Di Martino, A. Pisani	Interplay between endocannabinoids and dopamine in the basal ganglia: implications for pain in Parkinson's disease	2024	Assessment of the interactions between endocannabinoids and dopamine, the effects of endocannabinoids on ion and synaptic signaling in dopaminergic neurons. The impact of disrupted balance between endocannabinoids and dopamine on pain in Parkinson's disease.	review
E. Kaffe, A. Tisi, Ch. Magkrioti, V. Aidinis, W.Z Mehal, R. A Flavell, et al.	Bioactive signalling lipids as drivers of chronic liver diseases	2024	The role of lysophospholipids, eicosanoids, and endocannabinoids in the pathogenesis of chronic liver diseases	review
X. Di, B.M.Tellez, E. H J Krekels, L. Jurado-Fasoli, F. J Osuna-Prieto, L. Ortiz-Alvarez, et al.	Higher Plasma Levels of Endocannabinoids and Analogues Correlate With a Worse Cardiometabolic Profile in Young Adults	2023	Assessment of the relationship between plasma levels of endocannabinoids and their analogues with body composition and cardiometabolic risk factors<br><br>*Study result: Plasma levels of eCB and their analogues are associated with higher levels of obesity and a worse cardiometabolic profile.*	Clinical study. The study involved 133 young adults.

Y. Wu, Ch. Han, R. Luo, W. Cai, Q. Xia, R. Jiang, et al.	Molecular mechanisms of pain in acute pancreatitis: recent basic research advances and therapeutic implications	2023	The role of neuropeptides, ion channels, and the endocannabinoid system in pain related to acute pancreatitis	review
J.M.Quintero, L.Eduardo Diaz, I.Galve-Roperh, R.H. Bustos, M.X.Leon, S. Beltran, et al.	The endocannabinoid system as a therapeutic target in neuropathic pain: a review	2024	The role of the endocannabinoid system in regulating neuropathic pain and the therapeutic potential of cannabinoids	review
G. Vasudevan, K. Ramachandran, Ch. Tangavel, S. Miracle Nayagam, Ch. Gopalakrishnan, R. Muthurajan, et al.	Elucidating the immunomodulatory role of endocannabinoids in intervertebral disc degeneration	2024	Assessment of the immunomodulatory role of endocannabinoids in regulating intervertebral disc (IVD) health<br><br>*Study result: Endocannabinoid metabolites (2-AG and AEA) of the ECS were significantly lower in diseased discs compared to the control group.*	Clinical study The study involved 20 healthy volunteers and 40 patients with intervertebral disc degeneration.
A. Jana, A. Nath, P. Sen , S. Kundu, B. S Alghamdi, T. S. Abujamel, et al.	Unraveling the Endocannabinoid System: Exploring Its Therapeutic Potential in Autism Spectrum Disorder	2024	The role of the endocannabinoid system in the pathogenesis of neuropsychiatric disorders, including autism spectrum disorders	review
A. Pařízek, J. Suchopár, Z. Laštůvka, M. Alblová, M. Hill, M. Dušková	The Endocannabinoid System and Its Relationship to Human Reproduction	2023	The role of the endocannabinoid system in the proper functioning of reproductive organs	review
A. Pařízek, M. Hill, M. Dušková, L. Kolátorová, J. Suchopár, P. Šimják, et al	The Endocannabinoid System - The Prediction of Spontaneous Preterm Birth in High-Risk Women: Protocol of a Study	2023	Determination of the relationship between blood levels of studied endocannabinoids and the risk of spontaneous preterm birth (sPTB)	Clinical study. The study includes 230 women.<br><br>IN PROGRESS
Y.Fang Chen, Z.Kai Fan, Y.Peng Wang, P. Liu, X. Fei Guo, D. Li	Docosahexaenoic Acid Modulates Nonalcoholic Fatty Liver Disease by Suppressing Endocannabinoid System	2024	Determination of whether docosahexaenoic acid (DHA) supplementation alleviates non-alcoholic fatty liver disease (NAFLD) through the endocannabinoid system.<br><br>Study results: It was shown that serum levels of 2-AG and the expression level of the CB1 receptor in adipocytes were significantly lower in the	Clinical study. The levels of endocannabinoid ligands in serum were measured in 60 individuals with NAFLD and 60 healthy individuals. Mouse models

			DHA-supplemented group compared to the high-fat diet group.	were also used in the study.
E.Kyung Kwon, Y. Choi, S. Sim, Y.Min Ye, Y. Seob Shin, H.Sim Park, et al.	Cannabinoid receptor 2 as a regulator of inflammation induced oleoylethanolamide in eosinophilic asthma	2024	Assessment of the role of the CB2 receptor in eosinophil activation in vitro and in vivo.<br><br>Study results: The CB2 receptor may contribute to the pathogenesis of eosinophilic asthma.	Clinical study
N. Madnani, J.eztsna Deo, K. Dalal, B. Benjamin, V. V Murthy, R. Hegde, et al.	Revitalizing the skin: Exploring the role of barrier repair moisturizers	2024	Discussion on the role of moisturizers in repairing the protective barrier. The role of endocannabinoid mediators in maintaining a proper skin-epidermal barrier.	review
S. S Rathod, Y. O Agrawal	Phytocannabinoids as Potential Multitargeting Neuroprotectants in Alzheimer's Disease	2024	Assessment of the neuroprotective properties of phytocannabinoids and their impact on the regulation of the endocannabinoid system; resulting benefits in reducing Alzheimer's disease.	review
S. A More, R. S Deore, H. D Pawar, Ch. Sharma, K. T Nakhate, S. S Rathod, et al.	CB2 Cannabinoid Receptor as a Potential Target in Myocardial Infarction: Exploration of Molecular Pathogenesis and Therapeutic Strategies	2024	Assessment of the soothing potential of CB2 receptors in myocardial damage caused by various pathogenic mechanisms.	review
K. Crowley, Ł. Kiraga, E. Miszczuk, S. Skiba, J. Banach, U. Latek, et al.	Effects of Cannabinoids on Intestinal Motility, Barrier Permeability, and Therapeutic Potential in Gastrointestinal Diseases	2024	Assessment of the mechanisms of action and biological effects of endocannabinoids and phytocannabinoids on gastrointestinal functions and the potential therapeutic applications of these compounds.	review
P. Janiak, B. Poirier, J.-P. Bidouard, C. Cadrouvele, F. Pierre, L. Gouraud, et al.	Blockade of cannabinoid CB1 receptors improves renal function, metabolic profile, and increased survival of obese Zucker rats	2007	Assessment of the long-term treatment outcomes with the CB1 receptor antagonist rimonabant in chronic kidney failure associated with metabolic disorders.	Clinical study on rats.
F. Barutta, G. Bruno, R. Mastrocola, S. Bellini, G. Gruden	The role of cannabinoid signaling in acute and chronic kidney diseases	2018	Assessment of preclinical evidence indicating the role of the endocannabinoid system in kidney disease and discussion of potential future therapeutic applications.	review
A. Permyakova, A. Rothner, S. Knapp, A.	Renal Endocannabinoid Dysregulation in	2023	Assessment of changes in the endocannabinoid system in kidneys caused by obesity in	Clinical study. In total, the study was

Nemirovski, D. Ben-Zvi, J. Tam	Obesity-Induced Chronic Kidney Disease in Humans		humans. Study results: serum endocannabinoid levels were similar in lean and obese groups; endocannabinoid analysis in kidneys showed higher levels of anandamide in obese patients, who also exhibited decreased expression of cannabinoid receptor-1 in the kidneys, along with increased activity of enzymes synthesizing and degrading endocannabinoids.	conducted on a group of 21 obese and lean men.
F. Barutta, S. Grimaldi, R. Gambino, K. Vemuri, A. Makriyannis, L. Annaratone, et al.	Dual therapy targeting the endocannabinoid system prevents experimental diabetic nephropathy	2017	The role of the endocannabinoid system in the pathogenesis of diabetic nephropathy. Study results: "Peripheral" blockade of CB1R is beneficial in experimental diabetic nephropathy (DN), and this effect is synergistically enhanced by the activation of CB2R.	Clinical study on mouse models.
C. Zoja, M. Locatelli, D. Corna, S. Villa, D. Rottoli, V. Nava, et al.	Therapy with a Selective Cannabinoid Receptor Type 2 Agonist Limits Albuminuria and Renal Injury in Mice with Type 2 Diabetic Nephropathy	2016	Assessment of the impact of a CB2 agonist on functional and structural changes in the kidneys in type 2 diabetic nephropathy. Study results: Treatment with a CB2 agonist reduced progressive albuminuria and induced renoprotective effects in mice, similar to the effects of an ACE inhibitor.	Clinical study on mouse models.
L. Lecru, Ch. Desterke, S. Grassin-Delyle, Ch. Chatziantoniou, S. Vandermeersch, A. Devocelle, et al.	Cannabinoid receptor 1 is a major mediator of renal fibrosis	2015	The involvement of the CB1 receptor in the development of kidney fibrosis. Study results: the CB1 receptor plays a major role in the activation of myofibroblasts and may be a new target in the treatment of chronic kidney disease.	Clinical study on mouse models.
M.Dao, H. François	Cannabinoid Receptor 1 Inhibition in Chronic Kidney Disease: A New Therapeutic Toolbox	2021	Assessment of the therapeutic potential of the CB1 receptor in the context of chronic kidney disease.	review
H. Moradi, Ch. Park, E. Streja, D. A Argueta, N. V DiPatrizio, A. S You, et al.	Circulating Endocannabinoids and Mortality in Hemodialysis Patients	2020	Assessment of the relationship between serum 2-AG concentration and mortality in hemodialysis patients. Study results: Serum 2-AG concentration positively	Clinical study

			correlated with body mass index, serum triglyceride levels, and anthropometric body measurements. Meanwhile, serum AEA levels positively correlated with serum interleukin-6 and negatively with serum very low-density lipoprotein levels.	
A. N Friedman, J. Kim, Sh. Kaiser, Th. L Pedersen, J. W Newman, B. A Watkins	Association between plasma endocannabinoids and appetite in hemodialysis patients: A pilot study	2016	Assessment of the relationship between circulating endocannabinoids and appetite in hemodialysis patients.	Clinical study The study included a group of 20 patients.
P. Mukhopadhyay, M. Rajesh, H. Pan, V. Patel, B. Mukhopadhyay, S. Bátkai, et al.	Cannabinoid-2 receptor limits inflammation, oxidative/nitrosative stress, and cell death in nephropathy	2010	The role of CB2 receptors in cisplatin-induced nephrotoxicity. Study results: a CB2 receptor agonist attenuated cisplatin-induced inflammatory response, oxidative/nitrosative stress, and cell death in the kidneys, and improved kidney function, while CB2 knockout resulted in enhanced inflammation and tissue damage.	Clinical study on mouse models.
H. Moradi, F. Oveisi, E. Khanifar, G. Moreno-Sanz, N. D Vaziri, D. Piomelli	Increased Renal 2-Arachidonoylglycerol Level Is Associated with Improved Renal Function in a Mouse Model of Acute Kidney Injury	2016	The role of the endocannabinoid system in acute kidney injury from hypoperfusion (Ischemia-reperfusion injury (IRI)). Study results: A correlation was demonstrated between increased levels of 2-arachidonoylglycerol in the kidneys and improved kidney function in acute kidney injury from hypoperfusion.	Clinical study on mouse models.
J. Klawitter, C. Sempio, M. J Jackson, P. H Smith, K. Hopp, M. Chonchol, et al.	Endocannabinoid System in Polycystic Kidney Disease	2022	The role of the endocannabinoid system in the pathogenesis of ADPKD. Study results: compared to healthy individuals, patients with ADPKD had higher levels of interleukin-6 and -1b, as well as lower levels of anandamide, 2-arachidonoylglycerol, and their congeners in plasma.	The clinical study included a group of 102 patients and a group of 100 healthy volunteers.
